# The effect of pay-for-performance program on infection events and mortality rate in diabetic patients: a nationwide population-based cohort study

**DOI:** 10.1186/s12913-021-06091-2

**Published:** 2021-01-21

**Authors:** Yi-Fang Wu, Mei-Yen Chen, Tien-Hsing Chen, Po-Chang Wang, Yun-Shing Peng, Ming-Shyan Lin

**Affiliations:** 1 Department of Emergency Medicine, Chang Gung Memorial Hospital, Chiayi, Taiwan; 2 Department of Nursing, Chang Gung University of Science and Technology, Chiayi, Taiwan; 3 Department of Nursing, Chang Gung University, Taoyuan, Taiwan; 4 Department of Cardiology, Chang Gung Memorial Hospital, Keelung, Taiwan; 5 Biostatistical Consultation Center of Chang Gung Memorial Hospital, Keelung, Taiwan Community Medicine Research Center of Chang Gung Memorial Hospital, Keelung, Taiwan; 6 Chang Gung University, Taoyuan, Taiwan; 7 Department of Cardiology, Chang Gung Memorial Hospital, Chiayi, Taiwan; 8 Department of Endocrinology and Metabolism, Department of internal medicine, Chang Gung Memorial Hospital, Chiayi, Taiwan; 9 Graduate Institute of Clinical Medical Sciences, College of Medicine, Chang Gung University, Taoyuan, Taiwan

**Keywords:** Pay-for-performance, Infection, Sepsis, Diabetes, Mortality

## Abstract

**Background:**

Diabetes mellitus is a known risk factor for infection. Pay for Performance (P4P) program is designed to enhance the comprehensive patient care. The aim of this study is to evaluate the effect of the P4P program on infection incidence in type 2 diabetic patients.

**Methods:**

This is a retrospective longitudinal cohort study using data from the National Health Insurance Research Database in Taiwan. Diabetic patients between 1 January 2002 and 31 December 2013 were included. Primary outcomes analyzed were patient emergency room (ER) infection events and deaths.

**Results:**

After propensity score matching, there were 337,184 patients in both the P4P and non-P4P cohort. The results showed that patients’ completing one-year P4P program was associated with a decreased risk of any ER infection event (27.2% vs. 29%; subdistribution hazard ratio [HR] 0.87, 95% confidence interval [CI] 0.86–0.88). While the number needed to treat was 58 for the non-P4P group, it dropped to 28 in the P4P group. The risk of infection-related death was significantly lower in the P4P group than in the non-P4P group (4.1% vs. 7.6%; HR 0.46, 95% CI 0.45–0.47). The effect of P4P on ER infection incidence and infection-related death was more apparent in the subgroups of patients who were female, had diabetes duration ≥5 years, chronic kidney disease, higher Charlson’s Comorbidity Index scores and infection-related hospitalization in the previous 3 years.

**Conclusions:**

The P4P program might reduce risk of ER infection events and infection-related deaths in type 2 diabetic patients.

**Supplementary Information:**

The online version contains supplementary material available at 10.1186/s12913-021-06091-2.

## Background

Diabetes mellitus (DM) affected 463 million adults worldwide [1]. Its prevalence has increased over the past 10 years, reaching up to 9% of world population in 2019, with nearly 20% of patients being over 65 years old [1]. Diabetes is not only a metabolic disease, but it is also associated with multiple comorbidities, including chronic kidney disease (CKD), retinopathy, cardiovascular disease, stroke, and infections [2]. Over 10% of the global health expenditure is spent on diabetes treatment [3], with the total healthcare cost per diabetic patient being approximately 2.8 times higher than that of a non-diabetic patient [4].

Infection is the second leading cause of death in type 2 diabetes mellitus patients (T2DM), accounting for 10% of the annual emergency room (ER) visits and 12% of hospitalizations respectively [5, 6]. Diabetic patients are more likely than non-diabetic patients to develop sepsis, recurrent infections, hospitalization, shock, and mortality [7–9]. Therefore, there is a need for more medical interventions and more comprehensive diabetes care in order to diminish the risk of infection among T2DM patients [8].

Pay for Performance (P4P) program has been implemented in order to increase health care efficiency in many countries, including the United States, United Kingdom, Germany, and Taiwan [10]. Comparing with other diseases such as coronary heart disease and stroke, P4P program in DM had showed the highest rate of medical care quality improvement [11, 12]. The P4P program was initiated in Taiwan for enhancing diabetes care since November 2001 [13], more than one quarter of diabetic patients are enrolled in the program annually [14]. After patients’ enrollment in the P4P program, a team of care providers, consisting of physicians, nurses, nutritionists, and other healthcare professionals work together to provide serial examinations, health education, and follow-up services. Previous studies have showed that the P4P program, aimed at tackling diabetes, effectively increased clinical guideline adherence, quality of care, physician continuity, and decreased inpatient care utilization [13, 15–17].

Considering the improvement in diabetes care, we hypothesized that the P4P program will reduce infection-related complications and mortality rate in T2DM patients. Because previous research has smaller patient sample size and is limited on subgroup analysis of mortality and ER infections [18, 19], we conducted the present population-based study to address the relationship between infection associated outcomes and the diabetes P4P program.

## Methods

### Data source

This is a retrospective longitudinal cohort study using data from the Taiwan National Health Insurance Research Database (NHIRD). The National Health Institute (NHI) Program, launched in March 1995, provides 99% of medical coverage for the 23 million residents in Taiwan. The NHIRD claims information from the NHI program and is updated biannually and validated by the Taiwan’s Bureau of National Health Insurance (BNHI). The NHIRD contains data of outpatient and inpatient services, including diagnosis, medication, interventions, operations, hospitalizations, and emergent visits. Diagnosis is registered using the International Classification of Diseases, Ninth Revision, Clinical Modification (ICD-9-CM) codes. All patient information in the NHIRD is de-identified to protect privacy. For reference further information regarding NHI and NHIRD has been described in previous publications [20, 21]. The institutional review board of the Chang Gung Memorial Hospital approved this study (IRB No.:202000134B1).

### Pay for performance program in Taiwan

The diabetes P4P program was established in 2001 by the BNHI ([13], https://www.nhi.gov.tw/Content_List.aspx?n=95611DD9DDCAF987&topn=5FE8C9FEAE863B46). Patients who participated in the P4P program went through diet and health management education, annual laboratory tests (including HbA1c, fasting sugar, creatinine, alanine aminotransferase (ALT), total cholesterol, low-density lipoprotein (LDL), triglyceride (TG), high-density lipoprotein (HDL), and urinalysis), and serial physical examinations (ex. ophthalmic evaluation and fundoscopic exam) in order to enhance their comprehensive diabetes care [15, 22]. Diabetic patients were enrolled in the P4P program in Taiwan with payment coding as P1407C and received follow-up services every three months (P1408C). Patients who completed enrollment and more than two follow-ups in the first year went through an annual examination and were labeled as completing the first year P4P program (P1409C).

We used payment coding (P1409C) as completing the first year P4P program. Institutions (health care providers) that fulfilled the annual P4P requirements were qualified to receive financial support from the NHI. The health care providers are granted 650 NT dollars per qualified patient per physician, 400 NT dollars per new enrolled patient, 200 NT dollars per follow-up per patient and 800 NT dollars per qualified patient to the institution [21].

### Inclusion criteria and study design

**Fig. 1:**
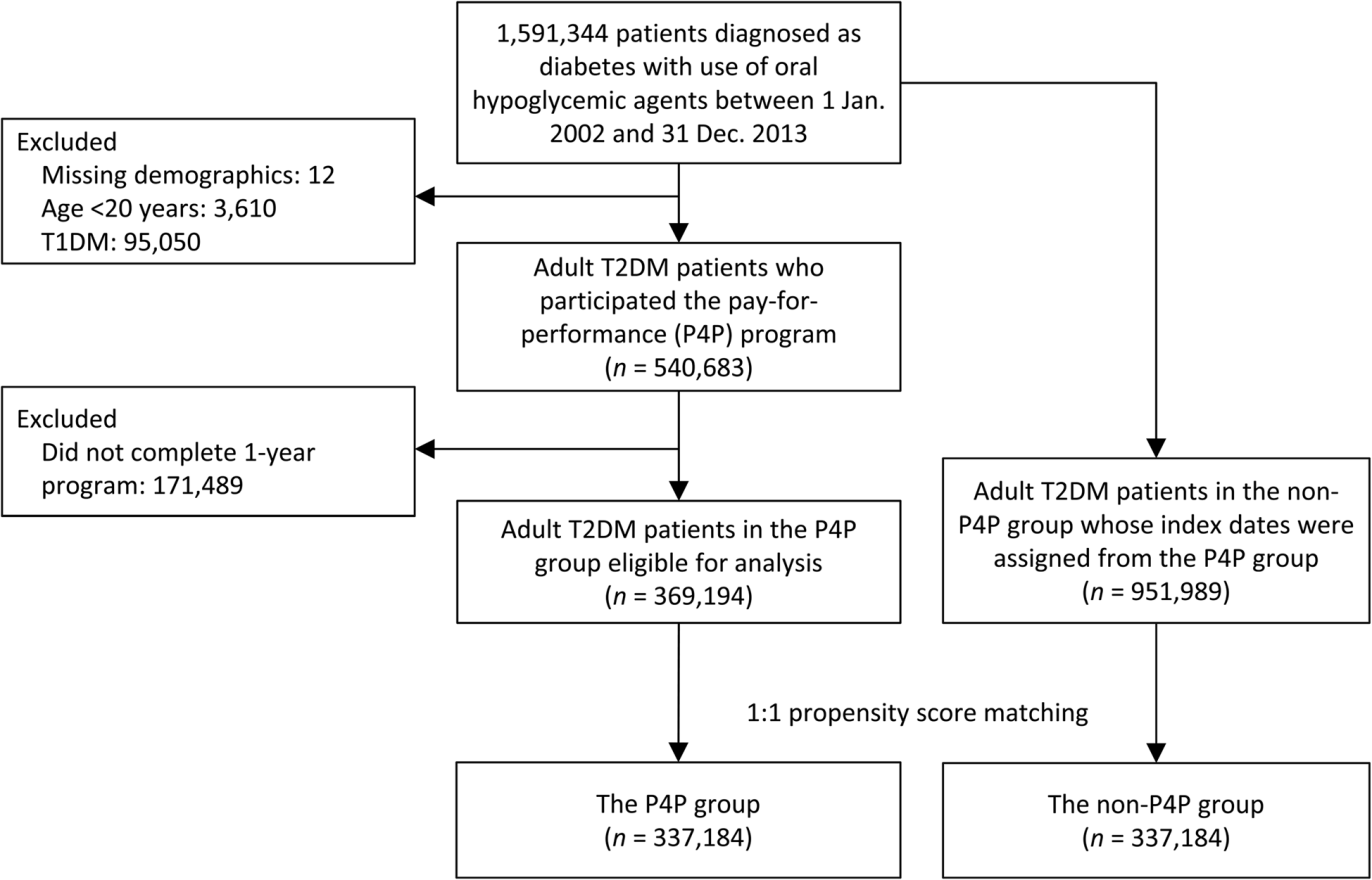
Patient selection. T1DM, type 1 diabetes mellitus; T2DM, type 2 diabetes mellitus; P4P, pay for performance

We identified patients who were diagnosed as diabetes with use of any oral hypoglycemic agents between 1 January 2002 and 31 December 2013 using data obtained from the NHIRD. Patients who were diagnosed as type 1 DM (T1DM), less than 20 years-old, and had missing demographic information were excluded from the analysis. Patients who participated in the P4P program but did not qualify for the **first-year program** were excluded. A total of 369,194 adult T2DM patients in the P4P group were eligible for analysis. The date of completing the **first-year program** was set as the index date of the P4P group. Adult T2DM patients who did not participate in the P4P program were selected as the control group (*n* = 951,989). To avoid the immortal time bias, the index date of the control group was assigned as the date on which the T2DM patients in the P4P group finished the **first-year program** [23]. The patients in the P4P group were propensity score matched to those in the non-P4P group with a 1:1 ratio, yielding 337,184 patients in either group (Fig. 1).

### Covariates

Covariates in this study were patents’ age at the index date, sex, urbanization level, monthly income, diabetes duration, health care utilization in the previous year (number of outpatient visit related to diabetes, outpatient visit, emergency room visit and hospitalization), twelve comorbidities, Charlson’s Comorbidity Index (CCI) score, history of cardiovascular events (heart failure, ischemic stroke, hemorrhage stroke), history of infection events (infection-related hospitalization in the previous year and number of infection-related hospitalization in the previous 3 years), intake of seven types of diabetes medication and other medications (Table 1). Comorbidities were identified as having at least 2 outpatient diagnoses or an inpatient diagnosis in the previous year. The ICD-9-CM diagnostic codes of the comorbidities are listed in Supplemental Table 1. Patients’ medical history was identified by looking at any inpatient diagnosis made before the index date. All the information regarding medication intake in the year before the index date were extracted from the claim data of outpatient visits or the refill requests for chronic illness medication in the pharmacy using the Anatomical Therapeutic Chemical codes or the Taiwan NHI reimbursement code.

**Table 1:** Baseline characteristics of patients according to completing 1-year pay-for-performance program or not

Variable	Data before matching			Data after matching		
	P4P (*n* = 369,194)	Non-P4P (*n* = 951,989)	STD	P4P (*n* = 337,184)	Non- P4P (*n* = 337,184)	STD
Age (years; mean ± SD)	60.9 ± 11.8	63.0 ± 13.5	− 0.17	61.0 ± 11.8	61.1 ± 12.9	< 0.01
Male	182,531 (49.4)	471,689 (49.5)	< 0.01	166,940 (49.5)	166,928 (49.5)	< 0.01
Urban level						
Low	41,918 (11.4)	113,792 (12.0)	−0.02	37,964 (11.3)	37,115 (11.0)	0.01
Moderate	121,999 (33.0)	295,819 (31.1)	0.04	110,255 (32.7)	109,306 (32.4)	0.01
High	113,856 (30.8)	279,267 (29.3)	0.03	103,690 (30.8)	103,591 (30.7)	< 0.01
Very High	91,421 (24.8)	263,111 (27.6)	−0.07	85,275 (25.3)	87,172 (25.9)	−0.01
Monthly income, NTD						
0–17,880	84,853 (23.0)	260,210 (27.3)	−0.10	78,630 (23.3)	78,457 (23.3)	< 0.01
17,881 – 22,800	151,438 (41.0)	374,004 (39.3)	0.04	137,209 (40.7)	136,259 (40.4)	0.01
> 22,800	132,903 (36.0)	317,775 (33.4)	0.06	121,345 (36.0)	122,468 (36.3)	−0.01
Diabetes duration (years)	5.8 ± 4.2	4.4 ± 3.8	0.36	5.5 ± 4.1	5.4 ± 4.1	0.03
Health care utilization in the previous year						
No. of outpatient visit of diabetes	14.4 ± 7.5	9.6 ± 8.1	0.62	13.8 ± 6.9	13.9 ± 8.7	− 0.02
No. of outpatient visit	34.1 ± 21.2	31.9 ± 22.8	0.10	33.5 ± 20.9	33.5 ± 21.7	< 0.01
No. of emergent room visit	0.37 ± 1.09	0.60 ± 1.81	− 0.16	0.37 ± 1.11	0.37 ± 0.99	0.01
No. of hospitalization	0.21 ± 0.67	0.50 ± 1.25	− 0.29	0.22 ± 0.68	0.22 ± 0.63	0.01
Comorbidity						
COPD	15,402 (4.2)	64,115 (6.7)	− 0.11	14,296 (4.2)	13,929 (4.1)	0.01
Hypertension	216,200 (58.6)	555,549 (58.4)	< 0.01	197,115 (58.5)	199,661 (59.2)	−0.02
PAOD	9503 (2.6)	22,965 (2.4)	0.01	8440 (2.5)	8237 (2.4)	< 0.01
Ischemic heart disease	48,383 (13.1)	151,326 (15.9)	−0.08	44,215 (13.1)	44,268 (13.1)	< 0.01
VTE	573 (0.16)	3600 (0.38)	−0.04	531 (0.2)	553 (0.2)	< 0.01
Dyslipidemia	198,296 (53.7)	331,620 (34.8)	0.39	174,589 (51.8)	177,147 (52.5)	−0.02
Auto-immune disease	1470 (0.40)	4537 (0.48)	−0.01	1332 (0.4)	1229 (0.4)	< 0.01
Liver disease	55,012 (14.9)	131,221 (13.8)	0.03	50,036 (14.8)	50,954 (15.1)	−0.01
Liver cirrhosis	4738 (1.3)	22,978 (2.4)	−0.08	4483 (1.3)	4338 (1.3)	< 0.01
Chronic kidney disease	62,472 (16.9)	118,749 (12.5)	0.13	52,110 (15.5)	49,095 (14.6)	0.03
Dialysis	987 (0.27)	20,406 (2.1)	−0.17	982 (0.3)	864 (0.3)	0.01
Malignancy	17,569 (4.8)	63,038 (6.6)	−0.08	16,312 (4.8)	16,194 (4.8)	< 0.01
Charlson’s Comorbidity Index score	2.1 ± 1.4	2.1 ± 1.9	0.03	2.1 ± 1.4	2.1 ± 1.6	< 0.01
History of event						
Heart failure	8910 (2.4)	56,558 (5.9)	−0.18	8391 (2.5)	8131 (2.4)	< 0.01
Ischemic stroke	23,773 (6.4)	112,059 (11.8)	−0.19	22,222 (6.6)	21,623 (6.4)	0.01
Hemorrhage stroke	2916 (0.79)	17,700 (1.9)	−0.09	2782 (0.8)	2593 (0.8)	0.01
History of infection event						
Infection-related hospitalization in the previous year	16,698 (4.5)	103,888 (10.9)	−0.24	15,856 (4.7)	15,119 (4.5)	0.01
No. of infection-related hospitalization in the previous 3 years	0.19 ± 0.62	0.42 ± 1.33	−0.22	0.19 ± 0.63	0.18 ± 0.63	0.02
Medication						
NSAID	71,133 (19.3)	192,047 (20.2)	−0.02	65,091 (19.3)	65,577 (19.4)	< 0.01
COX-II inhibitors	19,254 (5.2)	56,323 (5.9)	−0.03	17,609 (5.2)	17,709 (5.3)	< 0.01
Aspirin	91,631 (24.8)	209,685 (22.0)	0.07	81,838 (24.3)	81,656 (24.2)	< 0.01
Clopidogrel	5910 (1.6)	27,195 (2.9)	−0.09	5467 (1.6)	5309 (1.6)	< 0.01
Statin	149,217 (40.4)	231,072 (24.3)	0.35	128,993 (38.3)	128,449 (38.1)	< 0.01
Anticoagulant	2365 (0.64)	11,360 (1.2)	−0.06	2200 (0.7)	2170 (0.6)	< 0.01
Steroid	9995 (2.7)	38,984 (4.1)	−0.08	9267 (2.7)	9044 (2.7)	< 0.01
Antidiabetic medication						
Metformin	262,652 (71.1)	501,270 (52.7)	0.39	238,004 (70.6)	247,512 (73.4)	−0.06
DDP4 inhibitors	39,894 (10.8)	43,154 (4.5)	0.24	31,211 (9.3)	28,355 (8.4)	0.03
Sulfonylureas (SU)	228,801 (62.0)	476,608 (50.1)	0.24	208,035 (61.7)	215,388 (63.9)	−0.05
Thiazolidinedione	59,132 (16.0)	66,562 (7.0)	0.29	49,003 (14.5)	46,951 (13.9)	0.02
Non-SU insulin secretagogues	26,464 (7.2)	51,341 (5.4)	0.07	23,150 (6.9)	23,284 (6.9)	< 0.01
Alpha glucosidase inhibitors	50,046 (13.6)	72,429 (7.6)	0.19	42,604 (12.6)	42,000 (12.5)	0.01
Insulin	46,794 (12.7)	47,945 (5.0)	0.27	34,747 (10.3)	28,952 (8.6)	0.06
Follow-up (years; mean ± SD)	4.5 ± 3.0	3.5 ± 2.8	0.34	4.5 ± 3.0	3.9 ± 2.8	0.23

Abbreviations: *P4P* pay for performance; *STD* standardized difference; *SD* standard deviation; *NTD* new Taiwan dollar; *COPD* chronic obstructive pulmonary disease; *PAOD* peripheral artery occlusive disease; *VTE* venous thromboembolism; *DM* diabetes mellitus; *NSAID* non-steroidal anti-inflammatory drug; *COX-II* cyclooxygenase II; *DDP4* Dipeptidyl peptidase 4 inhibitors

Data were presented as frequency (percentage) or mean ± standard deviation

### Outcomes

The primary outcomes were patients’ first visit to the emergency room (ER) due to infection after the index date (any ER infection event) and infection-related death. Based on a previous study the infection sites included bacteremia, cardiovascular, central nervous system, respiratory, gastrointestinal (GI), genitourinary (GU), musculoskeletal and device-related infection (Supplemental Table 1) [24]. The infection-related death was determined by examining the cause of death in the main diagnosis in the discharge records for inpatient hospital deaths, the primary diagnosis of the last ER visit, or hospitalization within 7 days of death for out-of-hospital deaths [24]. Secondary outcomes were the number of all-cause ER visits, infection-related ER visits, all-cause hospitalization, infection-related hospitalization, and all-cause mortality during the follow up. In Taiwan, the most common reason for withdrawal from the NHI program was death, the other less common reasons include emigration or being missing more than 6 months. Therefore, a withdrawal from the NHI program was considered death in this study [25]. All patients were followed from the index date (the date of completing the first year P4P program for the P4P group) to the date of event, date of withdrawal from the NHI program or December 31, 2013, whichever came first.

### Statistical analysis

To reduce possible confounding of variables due to treatment selection-bias, PSM method was used in this study. The propensity score was the predicted probability of being included the P4P group given the values of covariates using the multivariable logistic regression without interaction effects. The variables selected to calculate the propensity score are listed in Table 1 where the follow up year was replaced with the index date. Each patient in the P4P group was matched with one counterpart in the non-P4P group. The matching was conducted using a greedy (nearest neighbor) algorithm with a caliper of 0.2 times of the standard deviation of the logit of propensity score, with random matching order and without replacement. The quality of matching was checked using the absolute value of standardized difference (STD) between the groups, where a value less than 0.1 was considered negligible difference.

The risks of time to fatal event outcomes (i.e., infection-related death and all-cause mortality) between the groups were compared using the Cox proportional hazard model. The time to non-fatal event outcomes (i.e., infection events) between groups were compared using the Fine and Gray subdistribution hazard model which considered all-cause mortality a competing risk. The difference in the number of events between the groups was compared using Poisson model in which the natural logarithm of follow up duration was treated as the offset variable. The study group was the only explanatory variable in the survival and Poisson models. The within-pair clustering of outcomes after PSM was accounted for by a robust standard error, which is known as the marginal model [26].

Subgroup analysis for any ER infection event and infection-related death were conducted on 6 pre-specified subgroup variables, including sex, age (20–39 years, 40–64 years and ≥ 65 years), diabetes duration (dichotomized by 5 years), CKD, CCI score (dichotomized by 2 scores) and infection-related hospitalization in the previous 3 years. A two-tailed *P* value < 0.05 was considered to be statistically significant and no adjustment for multiple testing (multiplicity) was made in this study. All statistical analyses were performed using SAS version 9.4 (SAS Institute, Cary, NC), including the “psmatch” procedure for PSM and the macro “%cif” for generating cumulative incidence function under the Fine and Gray subdistribution hazard method.

## Results

### Baseline patient characteristics

Table 1 lists the baseline characteristics of the patients included and not included in the P4P program before and after matching. Before matching, the P4P group was younger, had fewer ER visits and hospitalizations, lower prevalence of chronic obstructive pulmonary disease, dialysis, heart failure, stroke, and malignancy, but had longer diabetes duration, more outpatient health care utilization, higher prevalence of dyslipidemia and CKD. The two cohorts were similar in terms of the CCI score. After matching, there was no substantial difference (STD absolute value < 0.1) between the two groups. The mean follow-up duration was longer in the P4P group (4.5 years vs. 3.9 years).

### Outcomes

**Fig. 2:**
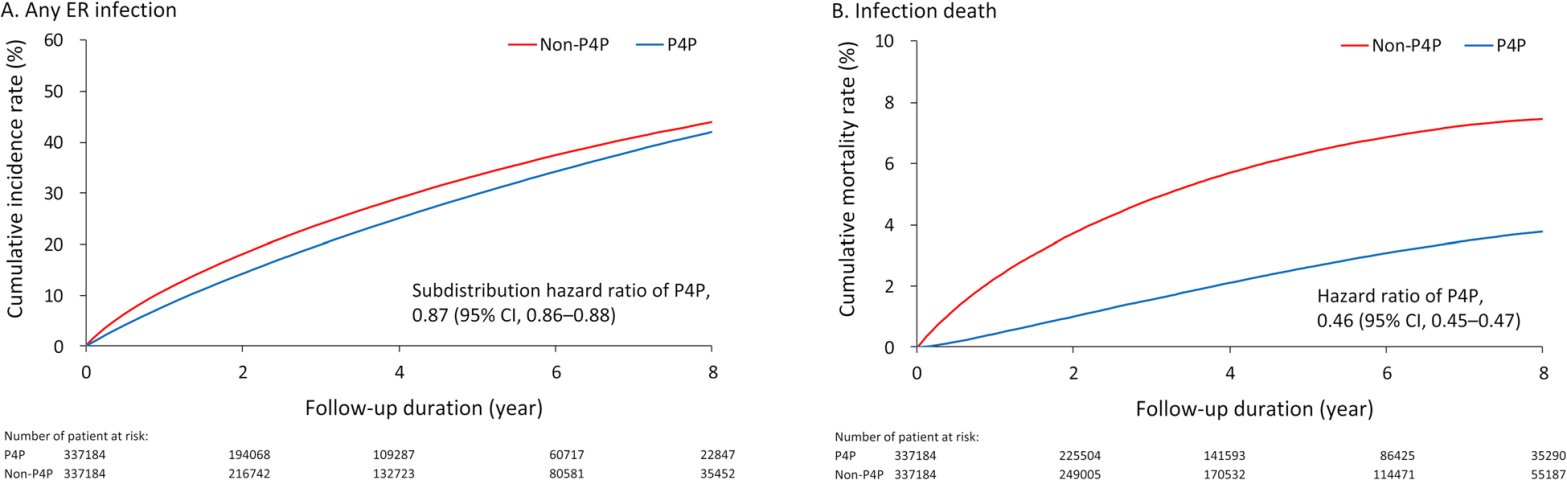
Cumulative incidence function under Fine and Gray method of any ER infection event (A) and cumulative event rates of infection death (B) of patients with or without one-year P4P program in the propensity score matched cohort

The results showed that completing the first year P4P program was associated with a decreased risk of any ER infection event (27.2% vs. 29%; subdistribution hazard ratio [HR] 0.87, 95% confidence interval [CI] 0.86–0.88) (Fig. 2a). The number needed to treat (NNT) was 58. According to the present results completing the first year P4P program may reduce the risks of all sites of ER infection events, except respiratory infection, with NNTs ranging between 36 and 5916. The risk of infection-related death was significantly lower in the P4P group than in the non-P4P group (4.1% vs. 7.6%; HR 0.46, 95% CI 0.45–0.47) with an NNT of 28 (Fig. 2b). In addition, completing the first year P4P program was associated with fewer events of all-cause ER visits, infection-related ER visits, all-cause hospitalization, and infection-related hospitalization during the follow up period. The risk of all-cause mortality was also significantly lower in the P4P group than in the non-P4P group (11.3% vs. 21.2%; HR 0.45, 95% CI 0.45–0.46, NNT = 10) (Table 2).

**Table 2:** Follow up outcomes in patients according to completing 1-year pay-for-performance program or not

Outcome	P4P (*n* = 337,184)	Non- P4P (*n* = 337,184)	RR, HR or SHR of P4P (95% CI)	NNT
ER visit due to infection				
Bacteremia	29,694 (8.8)	39,084 (11.6)	0.46 (0.45–0.47)	36
Cardiovascular	276 (0.08)	427 (0.13)	0.62 (0.53–0.72)	2233
Central nervous	656 (0.19)	713 (0.21)	0.88 (0.79–0.98)	5916
Respiratory	15,706 (4.7)	14,725 (4.4)	1.02 (0.997–1.04)*	344
Gastrointestinal	46,007 (13.6)	49,870 (14.8)	0.87 (0.86–0.88)	87
Genitourinary	54,131 (16.1)	57,449 (17.0)	0.89 (0.88–0.90)	102
Musculoskeletal	2581 (0.77)	2852 (0.85)	0.86 (0.82–0.91)	1244
Device-related infection	3432 (1.02)	3810 (1.13)	0.86 (0.82–0.90)	892
Any infection event	91,856 (27.2)	97,720 (29.0)	0.87 (0.86–0.88)	58
Infection death	13,882 (4.1)	25,752 (7.6)	0.46 (0.45–0.47)	28
Annual number of events during the follow up				
Number of all-cause ER visit	2.24 ± 4.88	2.04 ± 4.56	0.94 (0.93–0.95)	NA
Number of infection-related ER visit	0.40 ± 1.14	0.37 ± 1.07	0.91 (0.90–0.92)	NA
Number of all-cause hospitalization	1.49 ± 3.05	1.50 ± 2.92	0.85 (0.84–0.86)	NA
Number of infection-related hospitalization	0.48 ± 1.38	0.54 ± 1.40	0.76 (0.75–0.77)	NA
All-cause mortality	38,234 (11.3)	71,587 (21.2)	0.45 (0.45–0.46)	10

Abbreviations: *P4P* pay for performance; *CI* confidence interval; *RR* rate ratio; *HR* hazard ratio; *SHR* subdistribution hazard ratio; *CI* confidence interval; *ER* emergency room; *ICU* intensive care unit; *NNT* number needed to treat; *NA* not applicable;

Data were presented as frequency (percentage) or mean ± standard deviation;

* Not statistically significant

### Subgroup analysis

**Fig. 3:**
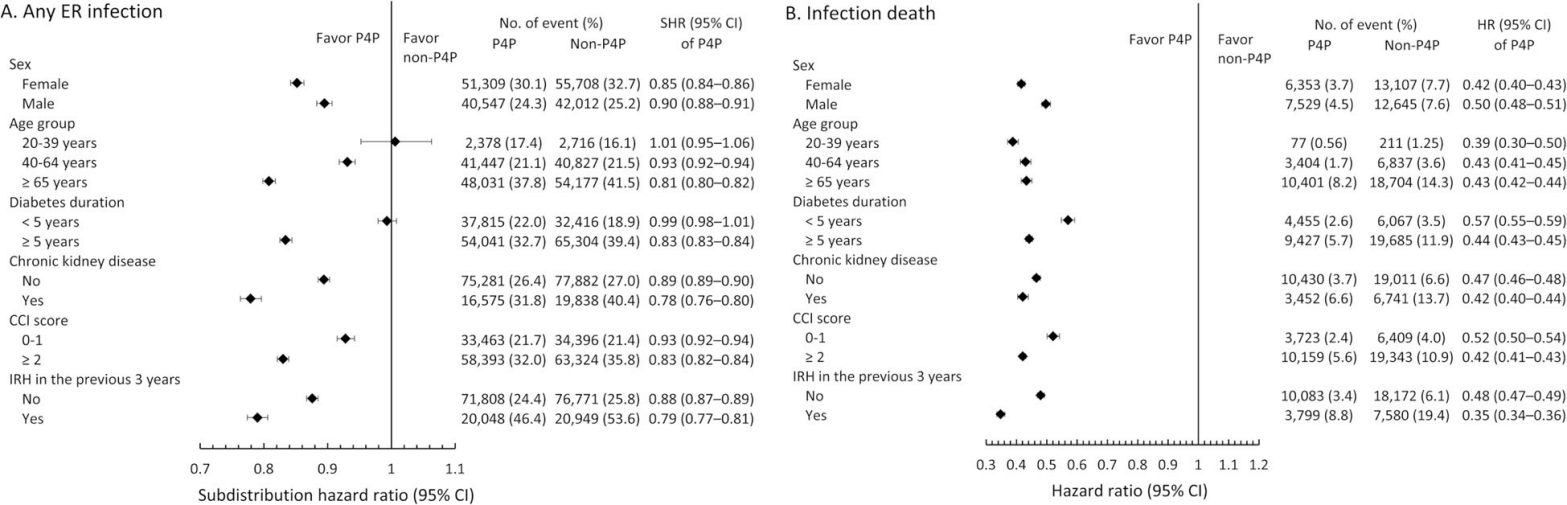
Pre-specified subgroup analysis of any ER infection event (A) and infection death (B)

We performed two subgroup analyses on the occurrence of all-cause ER infection events and infection-related deaths. The results suggested that the effect of P4P on ER infection events and infection-related deaths was more apparent in the subgroups of patients who were female, had diabetes duration ≥5 years, CKD, higher CCI scores and infection-related hospitalization in the previous 3 years. For patients aged ≥40 years old, risk reduction was more obvious for any ER infection events, but not for infection-related deaths (Figs. 3a-b).

## Discussion

This large population-based cohort analysis showed that the P4P program may significantly reduce (by 13%) the risks of ER infection events and infection-related hospitalization during the long-term follow up. Those benefits associated with a decreased risk of infection could improve the survival rate of the diabetic population and reduce the cost of medical health care through good care quality of P4P program [27].

Iain M. Carey et al. documented that T2DM patients who were aged > 70 years, obese, smoking, had longer diabetes duration, and living in more deprived areas had a higher risk of infection [7]. This is consistent with our study, which showed patients who had diabetes for more than 5 years, more than 40% of moderate urban level, higher ratio of CKD, and slightly higher proportion of insulin use. Those risk factors contribute to more diabetes-related complications, and how to optimize the comprehensive care was an essential issue. Under high-quality P4P program, serial diabetes-related tests and sustained improvement in continuity of care were both associated with lower risk of hospitalizations [4, 28], as well as early detection of unfavorable events including infections.

Urinary tract infection is the most common infection that accounted for 30% of ER visits in diabetic patients, followed by GI infection and bacteremia [6, 29]. Esper and his colleagues also documented that diabetic patients had a higher prevalence of GU infections (DM vs. non-DM 28% vs. 22%), and respiratory infections complicated with acute respiratory failure were significantly less frequent in the DM population [30]. In addition, they also found that diabetic patients with GU infections tended to suffer from acute renal failure, which might be a potential risk of mortality. Overall, those patients receiving P4P may get more survival benefit (reducing risk of infection-related death by 54% and all-cause mortality by 55%) through great risk reduction effect on the most common ER infections, including GU infection (16.1%, NNT = 102), followed by GI infection (13.6%, NNT = 87) and bacteremia (8.8%, NNT = 36).

All-cause mortality was noted to be almost twice as high for adults with T2DM than for the healthy adult population [31]. Major mortality causes other than infections included diseases of the circulatory system, respiratory system, endocrine, nutritional, and metabolic diseases [5]. Less literature discussed infection death among T2DM patients [18, 32]. In our study, the risk of infection-related death and all-cause mortality were both significantly lower in the P4P group than in the non-P4P group, which is consistent with previous investigations [16, 18, 32]. The P4P program was suggested to result in more frequent measures of HbA1C and set appropriate target of glycemic control [33]. High quality of diabetes care and adequate glycemic control could reduce macro- and micro-vascular complications, which both have been reported to affect outcomes of hospitalization [28, 29]. Poor glycemic control was not only related to higher risk of infection [27], but also higher mortality. HbA1c level > =6.5% was found to be a significant, independent predictor of severe organ dysfunction progression and mortality in sepsis [34]. Moreover, hypoglycemia with glucose level of less than 100 mg/dL was also associated with higher mortality [35, 36]. In our study, the cohort in P4P had a slightly higher percentage of insulin therapy, which was a risk factor for hypoglycemia and associated mortality. Although there is limited evidence on whether participation in the diabetes P4P program results in a better HbA1c level or not [33, 37], increased medication compliance and diet education in P4P may contribute to better glycemic stabilization and less risk of hypoglycemia, which could help to survive sepsis.

We noted the effect of P4P on ER infections and infection-related death was more apparent in female patients with diabetes duration ≥5 years, CKD, and higher CCI scores. A higher risk of infection-related hospitalization was noted in patients with longer DM duration and increased with patient’s age and components of multiple comorbidities in the research conducted by Iain M. Carey et al. [7]. Prolonged diabetes also contributed to leukocyte dysfunction, impaired immunity, anatomical abnormalities of the urinary tract and urinary dysmotility [38, 39]. Female diabetic patients had a higher incidence of GU infections in our study. Although sicker patients were usually excluded from the P4P program [22], those elder patients with longer DM duration, multiple comorbidities, and higher disease severity would potentially get more serious infection-related complications including shock and coma. For patients with more frequent infection-related hospitalization in the previous 3 years, P4P also showed a more risk reduction on recurrent ER infection events and potential infection-related deaths. Reduction of recurrent infections may result in a decrease in drug resistance bacteremia, improvement in the immune response, serious complications, and cost of hospitalization. The P4P program increased physician visits, serial exams and could raise medical expenses. However, the benefits outweigh costs due to expected reduction of ER visits and avoidance of infection-related complications during hospitalization.

Our study had several strengths including being a population-based study with a large sample size and long-term observation. However, we still had some inherent limitations caused by the retrospective analysis. First, Taiwan’s NHI Program is a single player system, which is different from health care systems in some other countries. The different program design could limit the application of the P4P program and demonstrate different effects in other regions. Second, the NHIRD limitations include the lack of records on self-paid healthcare and disease severity. We balanced the disease severity among both cohorts by collecting details of multiple comorbidities, medication intake and the CCI score. Third, the laboratory information including HbA1c and the glycemic level were not analyzed in this study. The effects of chronic glycemic control and glycemic control target on T2DM-associated infection need further studies. Fourth, the culture reports and antibiotic sensitivity were unviable in the analysis. However, the data might affect the duration of hospitalization and complications but was not associated with ER infection-related visit.

## Conclusions

In summary, our study showed that the diabetes P4P program may significantly decrease the risk of infection events, infection-related hospitalization, and mortality. The beneficial effects emerged to be more prominent in patients with longer diabetes duration, multiple comorbidities, and frequent previous infection-related hospitalization, indicating that enhancing multidisciplinary diabetes care on those sicker patients is clearly needed. However, we need further large, randomized control studies to confirm those findings.

## Supplementary Information

**Additional file 1 Supplemental Table 1.** ICD-9 CM diagnostic codes. Diagnosis is registered according to the International Classification of Diseases, Ninth Revision, Clinical Modification (ICD-9-CM) codes.

## Data Availability

The data used to support the findings of this study are available from the corresponding author upon request.

## Abbreviations

P4P: Pay for Performance
ER: emergency room
HR: Hazard ratio
CI: Confidence interval
DM: Diabetes mellitus
CKD: Chronic kidney disease
T1DM: Type 1 diabetes mellitus
T2DM: Type 2 diabetes mellitus
NHIRD: National Health Insurance Research Database
NHI: National Health Institute
BNHI: Bureau of National Health Insurance
ICD-9-CM: International Classification of Diseases, Ninth Revision, Clinical Modification
HbA1c: Glycated hemoglobin
ALT: Alanine aminotransferase
LDL: Total cholesterol, low-density lipoprotein
TG: Triglyceride
HDL: High-density lipoprotein
CCI: Charlson’s Comorbidity Index
GI: Gastrointestinal
GU: Genitourinary
STD: Standardized difference
PSM: Propensity score matching
NNT: Number needed to treat
NTD: New Taiwan dollar
COPD: Chronic obstructive pulmonary disease
PAOD: Peripheral artery occlusive disease
VTE: Venous thromboembolism
NSIAD: Non-steroidal anti-inflammatory drug
COX-II: Cyclooxygenase II
DDP4: Dipeptidyl peptidase 4
ICU: Intensive care unit
