# Supplementary material for: The effect of pay-for-performance program on infection events and mortality rate in diabetic patients: a nationwide population-based cohort study
Source: BMC Health Serv Res. 2021 Jan 21;21:78. doi: 10.1186/s12913-021-06091-2 (PMC7818736; doi:10.1186/s12913-021-06091-2)
Supplement: Supplementary file 1 — Additional file 1 Supplemental Table 1. ICD-9 CM diagnostic codes. Diagnosis is registered according to the International Classification of Diseases, Ninth Revision, Clinical Modification (ICD-9-CM) codes. [file 12913_2021_6091_MOESM1_ESM.docx]

**Supplemental Table 1**. ICD-9 CM diagnostic codes

| Variable | ICD-9 CM Code |
| --- | --- |
| Type 1 diabetes mellitus | 25001, 25003, 25011, 25013, 25021, 25023, 25031, 25033, 25041, 25043, 25051, 25053, 25061, 25063, 25071, 25073, 25081, 25083, 25091, 25093 |
| Type 2 diabetes mellitus | 250.xx (except for type 1 diabetes mellitus) |
| Diabetes mellitus | 250.xx |
| Chronic obstructive pulmonary disease | 491.xx, 492.xx, 496.xx |
| Hypertension | 401.xx-405.xx |
| Peripheral arterial disease | 440.0.xx, 440.2x, 440.3x, 440.8x, 440.9x, 443.xx, 444.0x, 444.22, 444.8x, 447.8x, 447.9x |
| Ischemic heart disease | 410.xx-414.xx |
| Venous thromboembolism | 453.xx, 415.1x |
| Dyslipidemia | 272.xx |
| Auto-immune disease | 7100, 7101, 7100, 7101, 7140, 7104, 7103, 4460, 4464, 4465, 4431, 4467, 4461, 1361, 6944, 7102, 555.xx, 556, 5560, 5561, 5562, 5563, 5564, 5565, 5566, 5568, 5569, 446.2x (Catastrophic illness card) |
| Liver disease | 070.xx, 456.0-456.2, 570.xx, 571.xx, 572.2-572.8, 573.xx, V42.7 |
| Liver cirrhosis | 571.2, 571.5, 571.6 |
| Chronic kidney disease | 580.xx-589.xx, 403.xx-404.xx, 016.0x, 095.4x, 236.9x, 250.4x, 274.1x, 442.1x, 447.3x, 440.1x, 572.4x, 642.1x, 646.2x, 753.1x, 283.11, 403.01, 404.02, 446.21 |
| Dialysis | 585.xx (Catastrophic illness card) |
| Malignancy | 140.xx-208.xx (Catastrophic illness card) |
| Heart failure | 428.xx |
| Ischemic stroke | 433.xx-437.xx |
| Hemorrhage stroke | 430.xx-432.xx |
| Infection-related hospitalization | 0031, 0362, 0380, 03810, 03811, 03812, 03819, 0382, 0383, 03840, 03841, 03842, 03843, 03844, 03849, 0388, 0389, 04082, 0545, 1125, 78552, 7907, 7908, 99591, 99592, 03282, 03640, 03641, 03642, 03643, 07420, 07421, 07422, 07423, 11281, 11503, 11504, 11593, 11594, 1303, 3910, 3911, 3912, 3918, 3919, 3920, 4210, 4211, 4219, 4220, 42292, 00321, 0360, 0361, 0470, 0471, 0478, 0479, 048, 0490, 0491, 0498, 0499, 0530, 05310, 05314, 0543, 05472, 05474, 0550, 05600, 05601, 05609, 05821, 05829, 0621, 0622, 0623, 0625, 0628, 0629, 0638, 0639, 064, 06641, 06642, 0721, 0722, 11283, 1142, 11501, 11591, 1300, 3200, 3201, 3202, 3203, 3207, 32081, 32082, 32089, 3209, 3210, 3211, 3212, 3230, 32301, 32302, 3231, 3234, 32341, 32342, 3240, 3241, 3249, 03283, 5670, 5671, 5672, 56721, 56722, 56723, 56729, 56789, 5679, 0030, 0038, 0039, 0040, 0041, 0043, 0048, 0049, 0050, 0051, 0052, 0053, 0054, 00581, 00589, 0059, 0071, 0074, 0075, 00800, 00801, 00802, 00803, 00804, 00809, 0081, 0082, 0083, 00841, 00842, 00843, 00844, 00845, 00846, 00847, 00849, 0085, 00861, 00862, 00863, 00864, 00865, 00866, 00867, 00869, 0088, 0090, 0091, 0092, 0093, 0392, 0700, 0701, 07043, 07053, 0723, 07271, 11285, 1305, 5400, 5401, 5409, 541, 542, 56201, 56203, 56211, 56213, 566, 56781, 5695, 5720, 5721, 5750, 57510, 03284, 0720, 59010, 59011, 5902, 5903, 59080, 59081, 5909, 5950, 5954, 59589, 5959, 5970, 59800, 59801, 5990, 6010, 6012, 6013, 6014, 6019, 6031, 6040, 60490, 60491, 6071, 6072, 6080, 6084, 6140, 6142, 6143, 6145, 6150, 6159, 6163, 6164, 00322, 01100, 01101, 01102, 01103, 01104, 01105, 01106, 01110, 01111, 01112, 01113, 01114, 01115, 01116, 01120, 01121, 01122, 01123, 01124, 01125, 01126, 01130, 01131, 01132, 01133, 01134, 01135, 01136, 01150, 01151, 01152, 01153, 01154, 01155, 01156, 01160, 01161, 01162, 01163, 01164, 01165, 01166, 01170, 01171, 01172, 01173, 01174, 01175, 01176, 01180, 01181, 01182, 01183, 01184, 01185, 01186, 01190, 01191, 01192, 01193, 01194, 01195, 01196, 0310, 0330, 0338, 0339, 0391, 0521, 0551, 0730, 0796, 1124, 1140, 1145, 11505, 11595, 1304, 1363, 4650, 4658, 4659, 4660, 46611, 46619, 4800, 4801, 4802, 4803, 4808, 4809, 481, 4820, 4821, 4822, 48230, 48231, 48232, 48239, 48240, 48241, 48242, 48249, 48281, 48282, 48283, 48284, 48289, 4829, 4830, 4831, 4838, 4841, 4843, 4846, 4847, 4848, 485, 486, 4870, 4871, 488, 4880, 4881, 490, 49122, 4941, 5100, 5109, 5111, 5130, 5131, 5192, 0311, 03285, 0390, 0400, 37601, 6800, 6801, 6802, 6803, 6804, 6805, 6806, 6807, 6808, 6809, 68100, 68101, 68110, 68111, 6819, 6820, 6821, 6822, 6823, 6824, 6825, 6826, 6827, 6828, 6829, 684, 6850, 6868, 6869, 72886, 9101, 9103, 9109, 9111, 9113, 9119, 9121, 9123, 9129, 9131, 9133, 9139, 9141, 9143, 9149, 9151, 9153, 9159, 9161, 9163, 9169, 9171, 9173, 9179, 9191, 9193, 9199, 00323, 00324, 03682, 37603, 05671, 71100, 71101, 71102, 71103, 71104, 71105, 71106, 71107, 71108, 71109, 71140, 71141, 71142, 71143, 71144, 71145, 71146, 71147, 71148, 71149, 71150, 71151, 71152, 71153, 71154, 71155, 71156, 71157, 71158, 71159, 71160, 71161, 71162, 71163, 71164, 71165, 71166, 71167, 71168, 71169, 71180, 71181, 71182, 71183, 71184, 71185, 71186, 71187, 71188, 71189, 71190, 71191, 71192, 71193, 71194, 71195, 71196, 71197, 71198, 71199, 73000, 73001, 73002, 73003, 73004, 73005, 73006, 73007, 73008, 73009, 73020, 73021, 73022, 73023, 73024, 73025, 73026, 73027, 73028, 73029, 73080, 73081, 73082, 73083, 73084, 73085, 73086, 73087, 73088, 73089, 73090, 73091, 73092, 73093, 73094, 73095, 73096, 73097, 73098, 73099, 99662, 99931, 99668, 53086, 53641, 56961, 99660, 99661, 99663, 99664, 99665, 99666, 99667, 99669, 99731, 99802, 99851, 99859, 9993, 99939 |
| Bacteremia | 0031, 0362, 0380, 03810, 03811, 03812, 03819, 0382, 0383, 03840, 03841, 03842, 03843, 03844, 03849, 0388, 0389, 04082, 0545, 1125, 78552, 7907, 7908, 99591, 99592 |
| Cardiovascular | 03282, 03640, 03641, 03642, 03643, 07420, 07421, 07422, 07423, 11281, 11503, 11504, 11593, 11594, 1303, 3910, 3911, 3912, 3918, 3919, 3920, 4210, 4211, 4219, 4220, 42292 |
| Central nervous | 00321, 0360, 0361, 0470, 0471, 0478, 0479, 048, 0490, 0491, 0498, 0499, 0530, 05310, 05314, 0543, 05472, 05474, 0550, 05600, 05601, 05609, 05821, 05829, 0621, 0622, 0623, 0625, 0628, 0629, 0638, 0639, 064, 06641, 06642, 0721, 0722, 11283, 1142, 11501, 11591, 1300, 3200, 3201, 3202, 3203, 3207, 32081, 32082, 32089, 3209, 3210, 3211, 3212, 3230, 32301, 32302, 3231, 3234, 32341, 32342, 3240, 3241, 3249 |
| Respiratory | 00322, 01100, 01101, 01102, 01103, 01104, 01105, 01106, 01110, 01111, 01112, 01113, 01114, 01115, 01116, 01120, 01121, 01122, 01123, 01124, 01125, 01126, 01130, 01131, 01132, 01133, 01134, 01135, 01136, 01150, 01151, 01152, 01153, 01154, 01155, 01156, 01160, 01161, 01162, 01163, 01164, 01165, 01166, 01170, 01171, 01172, 01173, 01174, 01175, 01176, 01180, 01181, 01182, 01183, 01184, 01185, 01186, 01190, 01191, 01192, 01193, 01194, 01195, 01196, 0310, 0330, 0338, 0339, 0391, 0521, 0551, 0730, 0796, 1124, 1140, 1145, 11505, 11595, 1304, 1363, 4650, 4658, 4659, 4660, 46611, 46619, 4800, 4801, 4802, 4803, 4808, 4809, 481, 4820, 4821, 4822, 48230, 48231, 48232, 48239, 48240, 48241, 48242, 48249, 48281, 48282, 48283, 48284, 48289, 4829, 4830, 4831, 4838, 4841, 4843, 4846, 4847, 4848, 485, 486, 4870, 4871, 488, 4880, 4881, 490, 49122, 4941, 5100, 5109, 5111, 5130, 5131, 5192 |
| Gastrointestinal | 03283, 5670, 5671, 5672, 56721, 56722, 56723, 56729, 56789, 5679, 0030, 0038, 0039, 0040, 0041, 0043, 0048, 0049, 0050, 0051, 0052, 0053, 0054, 00581, 00589, 0059, 0071, 0074, 0075, 00800, 00801, 00802, 00803, 00804, 00809, 0081, 0082, 0083, 00841, 00842, 00843, 00844, 00845, 00846, 00847, 00849, 0085, 00861, 00862, 00863, 00864, 00865, 00866, 00867, 00869, 0088, 0090, 0091, 0092, 0093, 0392, 0700, 0701, 07043, 07053, 0723, 07271, 11285, 1305, 5400, 5401, 5409, 541, 542, 56201, 56203, 56211, 56213, 566, 56781, 5695, 5720, 5721, 5750, 57510 |
| Genitourinary | 03284, 0720, 59010, 59011, 5902, 5903, 59080, 59081, 5909, 5950, 5954, 59589, 5959, 5970, 59800, 59801, 5990, 6010, 6012, 6013, 6014, 6019, 6031, 6040, 60490, 60491, 6071, 6072, 6080, 6084, 6140, 6142, 6143, 6145, 6150, 6159, 6163, 6164 |
| Musculoskeletal | 00323, 00324, 03682, 37603, 05671, 71100, 71101, 71102, 71103, 71104, 71105, 71106, 71107, 71108, 71109, 71140, 71141, 71142, 71143, 71144, 71145, 71146, 71147, 71148, 71149, 71150, 71151, 71152, 71153, 71154, 71155, 71156, 71157, 71158, 71159, 71160, 71161, 71162, 71163, 71164, 71165, 71166, 71167, 71168, 71169, 71180, 71181, 71182, 71183, 71184, 71185, 71186, 71187, 71188, 71189, 71190, 71191, 71192, 71193, 71194, 71195, 71196, 71197, 71198, 71199, 73000, 73001, 73002, 73003, 73004, 73005, 73006, 73007, 73008, 73009, 73020, 73021, 73022, 73023, 73024, 73025, 73026, 73027, 73028, 73029, 73080, 73081, 73082, 73083, 73084, 73085, 73086, 73087, 73088, 73089, 73090, 73091, 73092, 73093, 73094, 73095, 73096, 73097, 73098, 73099 |
| Device-related infection | 53086, 53641, 56961, 99660, 99661, 99663, 99664, 99665, 99666, 99667, 99669, 99731, 99802, 99851, 99859, 9993, 99939 |

ICD-9 CM, International Classification of Diseases, Ninth Revision, Clinical Modification.
